# In vitro and in vivo anti-colorectal cancer effect of the newly synthesized sericin/propolis/fluorouracil nanoplatform through modulation of PI3K/AKT/mTOR pathway

**DOI:** 10.1038/s41598-024-52722-z

**Published:** 2024-01-29

**Authors:** Shaimaa E. Diab, Nourhan A. Tayea, Bassma H. Elwakil, Salma S. Elshewemi, Abir Abd El Mageid Gad, Shaymaa A. Abdulmalek, Doaa A. Ghareeb, Zakia A. Olama

**Affiliations:** 1 Botany and Microbiology Department, Faculty of Science, Alexandria University, Alexandria, Egypt; 2 Medical Laboratory Technology Department, Faculty of Applied Health Sciences Technology, Pharos University in Alexandria, Alexandria, Egypt; 3 Zoology Department, Faculty of Science, Alexandria University, Alexandria, Egypt; 4 Applied Entomology Department, Faculty of Agriculture, Alexandria University, Alexandria, Egypt; 5 Bio-Screening and Preclinical Trial Lab, Biochemistry Department, Faculty of Science, Alexandria University, Alexandria, Egypt

**Keywords:** Cancer models, Cancer prevention, Cancer therapy, Nanoscale materials, Pharmacokinetics

## Abstract

The present work aimed to assess the potential effect of sericin/propolis/fluorouracil nanoformula against colorectal cancer (CRC) (the fourth most common cause of cancer-related mortalities). A novel anti-cancerous formula of the synthesized sericin/propolis nanoparticles was developed and tested both in vitro (using Caco-2 cell line) and in vivo (in experimentally induced colorectal cancer animal models). The combination index of the prepared nanoformula proved that the combination between sericin/propolis nanoparticles and 5-fluorouracil demonstrated the highest synergistic effect (0.86), with dose reduction index (DRI) of the chemotherapeutic drug reaching 1.49. The mechanism of action of the prepared nanoformula revealed that it acts through the inhibition of the PI3K/AKT/mTOR signaling pathway and consequently inhibiting cancerous cells proliferation. Treatment and prophylactic studies of both sericin and propolis showed increased TBARS (Thiobarbituric Acid Reactive Substance) formation, downregulated BCL2 (B-cell lymphoma 2) and activated BAX, Caspase 9 and Caspase 3 expression. The prepared nanoformula decreased the ROS (Reactive Oxygen Species) production in vivo owing to PI3K/AKT/mTOR pathway inhibition and FOXO-1 (Forkhead Box O1) activation that resulted in autophagy/apoptosis processes stimulation. The potent anticancer effect of the prepared nanoformula was further emphasized through the in vivo histopathological studies of experimentally induced tumors. The newly formulated sericin/propolis/fluorouracil nanoparticles exhibited clear-cut cytotoxic effects toward tumor cells with provided evidence for the prophylactic effect.

## Introduction

Approximately 9.4% of all cancer-related deaths in USA, Europe, northern and western Europe, and Australia were due to colorectal cancer (CRC)^1^. Colorectal cancers take a long time to develop, therefore there is a significant hope for secondary prevention^2–5^. Bevacizumab (a kind of anti-VEGF mAb) was the first angiogenesis inhibitor licensed for the treatment of colorectal cancer in 2004. It’s well known that a drug's therapeutic impact would be diminished as it gets eliminated by the body, hence the cancerous cells can quickly acquire resistance to many of these medications^6^. Therefore, tertiary prevention using chemoprophylaxis is the subject of recent studies which would increase the survival rate in the main and adjuvant therapy. Currently both chemotherapy options namely single agent, such as fluoropyrimidine, and combination chemotherapy regimens can be used in combating cancerous cells^7^.

On the other hand, natural products gained prominence as foundations of poly-pharmacological compounds for infectious illnesses, cancer, and neurological disorders^8^. Natural product research and development is ongoing, and approximately half of presently available cancer therapies originated directly or indirectly from natural substances^9^. These natural compounds can be of numerous forms, such as alkaloids, polysaccharides, polyphenols, diterpenoids, and unsaturated fatty acids. A synthetic flavonoid named Alvocidib was approved in phase 2 clinical studies as a treatment of various cancers including prostate, pancreatic cancer, and leukemias. Genistein, which is an isoflavonoid derived from soybeans is also approved in phase 2 clinical studies for the prevention and treatment of breast cancer and adenomas. Numerous other drugs derived from phenolic compounds with anticancer potentiality are all undergoing preclinical and clinical testing at the present time.

The continuous search for effective novel cancer treatments has expanded beyond studies of plant-based compounds. Researchers have examined the anticancer properties of honey^10^, propolis^11^, and venom peptides from wasps^12^ and ants^13^. *Apis mellifera* (propolis) is the most well researched and frequently employed bee product used for cancer prevention and therapy^14^. Moreover, Silk proteins are particularly interesting among the newly naturally based biopolymers that being proposed for biomedical applications because of their extraordinary properties such as processing versatility, oxygen and water vapor permeability, biocompatibility, enzymatic degradability, and diversity of side chain interactions available for 'decoration'^15^. Silk is predominantly obtained industrially from the genetically regulated silkworm *Bombyx mori*, which has very minimal batch-to-batch variability. Silk fibroin (SF, a fibrous protein) and silk sericin (SS, a globular protein) make up most of the silk fiber. Fibroin has been widely investigated and employed in a variety of biomaterial applications^15^. Silk sericin, which accounts for approximately 30% of the silk cocoon, belongs to a family of serine-rich silk proteins that bind fibroin fibers together^16^. For many years, as a byproduct of textile industry waste, SS was thrown in the degummed wastewater, producing a significant environmental impact. Previously it was, however, less researched than fibroin since some studies showed immunological and allergic reactions when present in the fiber in its original state^16^. Recent research has shown that the extracted SS does not exhibit immunogenic characteristics, making it a suitable biopolymer for biomedical and biotechnological applications. SS received a lot of attention in the literatures because of its biodegradability, biocompatibility, antioxidant activity, and regeneration potentiality^16,17^.

The aim of the present work was to newly synthesize sericin/propolis loaded nanoformula with the chemotherapeutic drug 5-Fluorouracil (5-FLU). Moreover, the novel nanoformula efficacy as a potent prophylactic and therapeutic agent against colorectal carcinoma were assessed in vitro and in vivo to elaborate the underlying mechanism. We used in vitro study just to choose the most effective and safe preparation that had the highest therapeutic effect with highest efficacy in vitro then examined it’s prophylactic and therapeutic effect in vivo.

## Materials and methods

### Materials

Sericin *Bombyx mori* (silkworm) (S5201-1G, MW 75 kDa) and 5-Fluorouracil (F0250000) were purchased from Sigma-Aldrich. Propolis description and analyses were previously reported in Elwakil et al.^18^. Caco-2 cells (ATCC HTB-37) were provided from American Type Culture Collection.

### Study approval

All animal experiments were done according to the approval ethics that obtained from the Care and Use of Laboratory Animals approved by the Institutional Animal Care and Use Committees (IACUCs) of Faculty of Science, Alexandria University, and was in accordance with ARRIVE guidelines and the International Standards for the Care and Use of Laboratory Animals of the European Community Directive of 1986; AU 04/23/01/25/2/01. Under optimum conditions of temperature, light and humidity, animals were housed in standard plastic cages and allowed for free access to pellet chow with water ad libitum. Animals spent 1 week (the period of adaptation) in a fixed condition before starting the experiment.

### Sericin/propolis nanoparticles (nSE/PRO) preparation and characterization

Sericin/propolis nanoparticles (nSE/PRO) were prepared according to Diab et al.,^19^ by adjusting different sericin to propolis ratio plus the stirring time of the reaction mixture. The suspensions attained were subjected to Zetasizer primitive characterization (Malvern Zetasizer Nano ZS, UK) to confirm the nanoparticles synthesis and stability^19^. Various Fluorouracil concentrations (10, 20 and 30 mg/ml) were added to the optimum trial then the combination index (CI) was determined. Moreover, to assess the shape and size of the prepared nanoparticles, transmission electron microscope (TEM, JEM-100 CX Joel) study was conducted. Further analysis was conducted through Fourier-transform infrared spectroscopy (FTIR).

### The in vitro anticancer effect of sericin/propolis nanoparticles (nSE/PRO) on colorectal cell lines (Caco-2 (ATCC HTB-37)).

#### IC50 determination

IC50 was calculated according to Shahin et al.^20^ in which it was reported different concentrations of each nSE/PRO trial were added, and cell viability was measured after 24 h using neutral red uptake assay. The extracted neutral red color intensity was measured at 490 nm in a microtiter plate reader spectrophotometer.

#### Anticancer mechanism of the most promising nSE/PRO nanoparticles’ formulations.

Caco-2 cells (ATCC HTB-37) (1 × 10^6^ cell/mL) were inoculated and cultured into a six-well plate after being treated with IC50 concentration of the most promising trials. Trypsin/EDTA solution was added (at 37 °C for 2 min) to detach the cells after media aspiration. The cells were collected and resuspended in 1 mL PBS and divided into two portions, one for RNA isolation and the second for homogenate preparation^20^.

### Cancer’s animal model

The present study was carried out using 105 in bred- albino mice; normal health and immune status; (25 ± 5 g) obtained from Pharmaceutical and Fermentation Industries Development Centre (PFIDC), The City of Scientific Research and Technological Applications (SRTA-City), Egypt. The randomized-housed mice were maintained at standard temperature, humidity, and a 12-h light–dark cycle. Free food and water were served. The acclimation period was one week before the beginning of the experiment. The experiment was performed in accordance with the Animal Care and Use Committee at the Faculty of Science, Alexandria University, and was in accordance with the ARRIVE guidelines and the International Standards for the Care and Use of Laboratory Animals of the European Community Directive of 1986; AU 04/23/01/25/2/01. Colorectal carcinoma induction or positive control group was done through 20 mg/kg BW of 1,2-Dimethyl hydrazine (DMH) intraperitoneally injected once a week for 8 consecutive weeks. Mice were divided into:

**(A) Prophylactic groups** (45 mice, prophylactic agents received orally for 8 weeks followed by cancer induction for 8 weeks; the experiment duration was 16 weeks).

- Group 1: Negative control (normal) (7 mice).
- Group 2: Sham control (no treatment—oral administrated saline) for 16 weeks (7 mice).
- Group 3: Positive control (10 mice) orally administered saline for 8 weeks then followed by DMH IP injection for addition 8 weeks.
- Group 4: SER prophylactic (Prophylactic sericin) group, mice received 75 mg sericin/Kg BW by gastric gavage for 8 weeks followed by carcinogenic agent injection for additional 8 weeks, (7 mice)
- Group 5: PRO prophylactic (Prophylactic propolis) group, mice received 75 mg propolis/Kg BW by gastric gavage for 8 weeks followed by carcinogenic agent injection for additional 8 weeks, (7 mice)
- Group 6: nSER/PRO prophylactic group, mice received 75 mg sericin/propolis nanoparticles/Kg BW by gastric gavage for 8 weeks followed by carcinogenic agent injection for additional 8 weeks (7 mice).

Three mice from positive control group were randomly selected and euthanized by isoflurane overdose inhalation then colon histology was done to confirm the presence of cancer before the experiment end.

**(B) Cancer treatment groups** (cancer induction followed by treatment; 59 mice, the experiment duration was 16 weeks).

- Group 1: Negative control (normal) (7 mice).
- Group 2: Sham control (no treatment—oral administrated saline) for 16 weeks (7 mice).
- Group 3: Positive control (10 mice) was IP injected with DMH for 8 weeks followed by oral administration of saline for 8 weeks.

Three mice from positive control group were randomly selected after 8 weeks of induction and euthanized by isoflurane overdose inhalation then colon histology was done to confirm the presence of cancer before the starting of treatment protocols.

- Group 4: carcinogenic agent injection started and continued for 8 weeks followed by oral treatments for additional 8 weeks. Group 4 was Subdivided into:
  - Group 4I, mice received 75 mg sericin/Kg BW by gastric gavage for 8 weeks after cancer induction (7 mice).
  - Group 4II, mice received 75 mg propolis/Kg BW by gastric gavage for 8 weeks after cancer induction (7 mice).
  - Group 4III, mice received 75 mg sericin/propolis nanoparticles/Kg BW by gastric gavage for 8 weeks after cancer induction (7 mice).
  - Group 4IV, mice received 75 mg SER/PRO/FLU/Kg BW by gastric gavage for 8 weeks after cancer induction (7 mice).
  - Group 4 V, mice received 75 mg FLU/Kg BW by gastric gavage for 8 weeks after the cancer was induced (7 mice).

**Figure 1:**
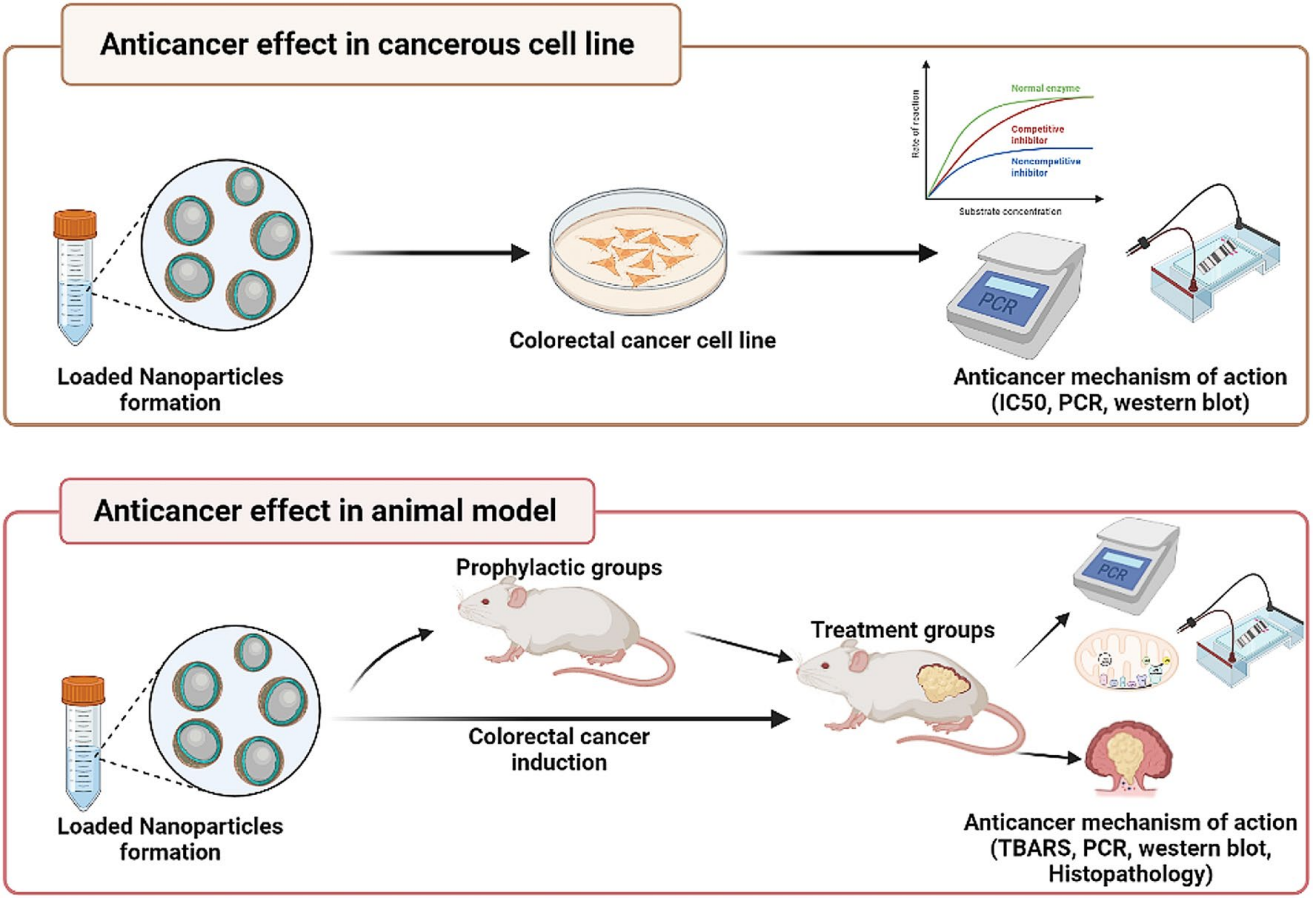
Study design.

After 16 weeks of treatments, all mice groups were euthanized by isoflurane overdose inhalation and necropsied. Colon tissue was collected and divided into three sections: one for histological assessments and the other parts were used for biochemical and molecular investigations. For histological study, colon tissue (part one) was fixed in 10% neutral buffered formalin (*n* = 3/group). At the same time, the second section of the colon tissue was stored at -80 °C for molecular analysis. The third Sect. 10% (w/v) was homogenized in phosphate buffer saline (0.1 M, pH 7.4), centrifuged at 10,000 rpm, 4 °C for 20 min, and the supernatant was utilized to assess the biochemical parameters (Fig. 1).

### Biochemical and Molecular investigations

#### Prooxidant determination

Colon tissue malondialdehyde (MDA) level was assessed in terms of thiobarbituric acid (TBA) reaction, according to Ghareeb et al.^21^.

#### RNA extraction and amplification

The centrifuged Caco-2 -treated cells or colon-isolated tissue was subjected to RNA isolation by using RNA isolation kit (iNtRON Biotechnology, Korea) according to manufacture instruction and according to Khazaei et al.^22^. RNA concentration and purity was measured at A260 and A260/A280 nm, respectively, using a Thermo ScientificNanoDrop™1000 Spectrophotometer along with its analytical software V3.7 (Thermo Fisher Scientific, DE, USA). Quantitative PCR was performed on reference and target genes (Table 1). RNA was converted into cDNA by using sensiFAST cDNA synthesis kit (Bioline, London). The gene amplification was carried out by using qPCR in which β-actin acts as a housekeeping gene. In PCR tube, 12.5 µL sensiFAST SYBR (Bioline, London) were mixed with 1µL of cDNA, 0.5 µL of 10 pmol/mL forward and reverse primers for each tested gene (Table 1). Then the volume was completed to 20 µL with nuclease-free distilled water. Samples were placed in the cycler and start the program as follows; 1 cycle of 95 °C for 10 min (initial denaturation), followed by 40 cycles of 95 °C for 15 s (denaturation), 60 °C for 30 s (annealing) and 72 °C for 30 s (extension) using a CFX96™ Real-Time System (BIO-RAD, USA). The critical threshold (Ct) quantities of the target gene were normalized with the house- keeping gene (β-actin) quantities by using the 2^−ΔΔCt^ method to calculate the fold change in the target genes.

**Table 1:** Primers’ sequences of target genes in vitro and in vivo.

Study	Tested gene	Primer sequence
In vitro	P53	F: CCTCAGCATCTTATCCGAGTGG
		R: TGGATGGTGGTACAGTCAGAGC
	PI3K	F: GGTTGTCTGTCAATCGGTGACTGT
		R: GAACTGCAGTGCACCTTTCAAGC
	BCL-2	F: ATCGCCCTGTGGATGACTGAGT
		R: GCCAGGAGAAATCAAACAGAGGC
	mTOR	F: GCTTGATTTGGTTCCCAGGACAGT
		R: GCTTGATTTGGTTCCCAGGACAGT
	AKT	F: TTCTGCAGCTATGCGCAATGTG
		R: TGGCCAGCATACCATAGTGAGGTT
	PGC-1α	F: CCAAAGGATGCGCTCTCGTTCA
		R: CGGTGTCTGTAGTGGCTTGACT
	VEGF	F: TTGCCTTGCTGCTCTACCTCCA
		R: GATGGCAGTAGCTGCGCTGATA
	PTEN	F: GGTTGCCACAAAGTGCCTCGTTTA
		R: CAGGTAGAAGGCAACTCTGCCAAA
	β-actin	F: CACCATTGGCAATGAGCGGTTC
		R: AGGTCTTTGCGGATGTCCACGT
In vivo	Caspase 3	F: GTGGAACTGACGATGATATGGC
		R: CGCAAAGTGACTGGATGAACC
	Caspase 9	F: AGTTCCCGGGTGCTGTCTAT
		R: GCCATGGTCTTTCTGCTCAC
	Bax	F: CGGCGAATTGGAGATGAACTGG
		R: CTAGCAAAGTAGAAGAGGGCAACC
	BCL-2	F: TGTGGATGACTGACTACCTGAACC
		R: CAGCCAGGAGAAATCAAACAGAGG
	β-actin	F: AAGATCCTGACCGAGCGTGG
		R: CAGCACTGTGTTGGCATAGAGG

#### Western blot

Radioimmunoprecipitation assay buffer (RIPA, 10 mM Tris–HCl (pH 7.4), 0.1% SDS, 150 mM NaCl, 1 mM EDTA, 1% Triton X-100, and 0.1% protease inhibitor cocktail) was used to homogenize the cell pellet or the colon tissue, then supernatants were collected. The supernatant protein concentration was assessed. The protein samples were run in 12% SDS-PAGE, then transferred to nitrocellulose membrane, and incubated overnight at 4 °C with primary antibodies namely Beclin 1, LC3, ERK, PERK and β-actin. The membranes were washed and incubated in secondary antibody (goat anti-rabbit IgG antibody ALP, 1:10,000) overnight at 4 °C, washed again after incubation then finally subjected to Alkaline phosphatase chromogen Substrate Kit (BCIP/TNBT). The intensities of bands obtained from western blot were estimated using image-analyzing system (UVitec software)^23^.

#### Histopathological examination

The fixed colon tissue was washed, dehydrated in serial dilutions of alcohol (methyl, ethyl and absolute ethyl alcohol). Specimens were embedded in paraffin then tissue blocks were sectioned using microtome (4 microns thickness). The obtained tissue sections were deparaffinized and stained by hematoxylin and eosin stain for routine examination^21^.

#### Acute toxicity and Pharmacokinetic studies of different nanoformula

### Acute toxicity study

Thirty-five male rats weighting about 200–220 g were raised in 25 °C, 25% humidity, in the Animal Center of the center for drug, pharmaceutical and fermentation industry development, SRTA-City under animal ethical guidelines. Rats used in this experiment were adapted firstly, forbidden to eat for 10 h before the experiment. Rats were divided in to five groups as follow:

- Control group (7 rats)
- Oral administration group: divided in to 4 subgroups, each subgroup formed from 7 rats and treated with different doses (10 mg/Kg, 100 mg/Kg, 1 g/Kg, 10 g/Kg).

After two weeks, dead rats were counted to calculate LD 50.

### Pharmacokinetic study of the newly prepared nano-formulae

Fourteen male rats weighting about 200–220 g were raised in 25 °C, 25% humidity, in the Animal Center of the center for drug, pharmaceutical and fermentation industry development, SRTA-City under animal ethical guidelines.

The rats were forbidden to eat for one hour before the experiment. Rats were divided in to two groups each contained 7 rats as follows:

- Negative Control group (7 rats)
- Oral administration group: dose 10 mg/Kg (7 rats)

**Figure 2:**
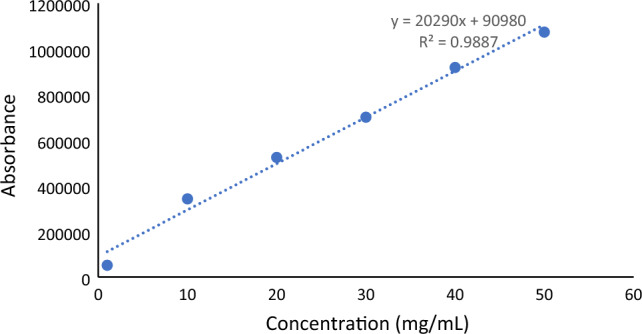
Fluorouracil standard curve.

Blood samples withdrawal at different time intervals per hours after the oral administration (0.5, 1, 2, 4, 6, 12 and 24 h). Rats were sacrificed after 24 h for pharmacokinetics investigations. The HPLC system (Shimadzu HPLC, Kyoto, Japan) that used in this experiment was equipped with UV–Vis detector at 254 nm, a system controller, a reverse-phase C18 column (150 × 4.6 mm) and autoinjector. The concentration of 5-FLU in the collected serum was determined using the method reported by Rahman et al.,^24^. The mobile phase was methanol, acetic acid and water (4: 0.05: 95.95, by volume), the flow rate was 1.0 mL min^−1^. 5-FLU was extracted from 0.5 mL serum by 0.5 mL of 10% v/v perchloric acid, the mixture was vortexed for 2 min and centrifuged at 2000 g for 10 min. The supernatant was filtered through a 0.2 μm membrane then 20 μL of the test sample was injected onto the column. The retention time of 5-FLU was 7 min. Standard curve of 5-FLU was conducted in rage of 1–50 μg mL^−1^ (Fig. 2).

### Statistical analysis

The number of animals was calculated according to a previous study^25^. Taking into account that the differences in the tested groups achieved an effect size of *d* = 1.76, assuming a sample size ratio of 1 and a statistical power (1 − *β* = 0.8) to identify significant differences (*α* = 0.05), 7 animals per group were necessary^25^. Each variable was tested for normality and variance using the Kolmogorov–Smirnov and Levene tests, respectively. Data are presented as means ± standard deviation. Hypothesis testing method included one-way analysis of variance (ANOVA) followed by least significant difference (LSD) test and Tukey post hoc test where p values of less than 0.05 were considered to indicate statistical significance.

### Ethical approval

The present experiment was performed in accordance with the Animal Care and Use Committee at the Faculty of Science, Alexandria University, and was in accordance with the ARRIVE guidelines and the International Standards for the Care and Use of Laboratory Animals of the European Community Directive of 1986; AU 04/23/01/25/2/01.

## Results

### Sericin/Propolis Nanoparticles (nSE/P) Preparation

In a trial to test the successful sericin/propolis nanoparticles’ formation, different sericin concentrations and stirring times of the reaction mixture were investigated. Table 2 showed that the best preparations with promising cytotoxic effects toward Caco-2 were 4, 15, 16 and 20. Moreover, it was revealed that the zeta potential ranged between 20.5 and 40.4 mV indicating the stability of the prepared nanoparticles. The observed zeta size of the most potent trials 4, 15, 16 and 20 were 116, 362.9, 269.9 and 222.6 nm respectively. The lowest IC50 (23.68 µg/ml) was recorded in trial 20 with sericin/propolis ratio 2/1 and stirring time 60 min. It is worth noting that by increasing the sericin ratio in the prepared formula, higher anticancer activity in vitro was reported.

**Table 2:** Anticancer activity of the synthesized nanoformulae against colorectal (Caco-2) carcinoma.

Trial number	Sericin/propolis ratio	Stirring time (min)	IC50 Concentration (µg/ml)	Physical characteristics		
				Zeta Potential (mV)	Size (nm)	PDI
1	1/1	15.0	94.40 ± 5.9	25.5	7540.0	0.27
2	1/1	30.0	53.14 ± 1.3	27.5	629.6	0.28
3	1/1	45.0	51.08 ± 0.9	35.6	166.1	0.65
4	1/1	60.0	32.09 ± 1.1	39.3	116.0	1.00
5	2/3	15.0	99.50 ± 6.7	28.6	1438.0	0.45
6	2/3	30.0	98.80 ± 3.7	33.3	642.3	0.47
7	2/3	45.0	87.80 ± 2.4	33.4	389.6	0.75
8	2/3	60.0	80.40 ± 10.7	40.4	143.2	0.97
9	1/2	15.0	284.24 ± 11.4	22.7	394.4	0.39
10	1/2	30.0	198.50 ± 12.3	23.0	362.8	0.41
11	1/2	45.0	184.04 ± 6.7	25.7	319.1	0.56
12	1/2	60.0	105.19 ± 0.8	27.2	270.3	0.46
13	3/2	15.0	47.38 ± 0.8	20.5	617.3	0.27
14	3/2	30.0	39.06 ± 1.9	23.0	375.0	0.31
15	3/2	45.0	35.01 ± 0.6	23.6	362.9	0.33
16	3/2	60.0	29.80 ± 1.7	24.8	269.9	0.36
17	2/1	15.0	68.20 ± 1.6	24.4	269.3	0.22
18	2/1	30.0	49.16 ± 2.4	28.2	268.0	0.24
19	2/1	45.0	47.20 ± 1.7	29.7	234.0	0.26
20	2/1	60.0	23.68 ± 2.6	32.7	222.6	0.30

### In vitro* molecular investigations*

**Figure 3:**
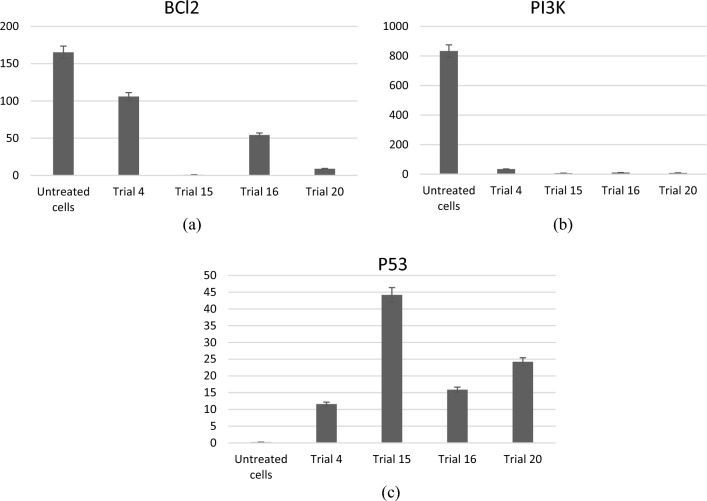
BCl2 (**a**), PI3K (**b**) and P53 (**c**) expression level upon using trials 4, 15, 16 and 20.

Table 3 proved that phosphatidylinositol (PI)-3-kinase/protein kinase/mammalian targeted of rapamycin (PI3K/AKT/mTOR) signaling pathway is active in cancer cells. All treatments inhibited PI3K/AKT/mTOR signaling pathway and prevented cell proliferation and angiogenesis (Fig. 3). The results indicated that the most powerful inhibitor for Caco-2 was preparation # 15 followed by 20 then 16 and 4 respectively hence trail 15 was selected for further experiments.

**Table 3:** Effect of different treatments on PI3K/AKT/mTOR signaling pathway gene expression of Caco-2.

Tested gene	Untreated cells	Trial 4	Trial 15	Trial 16	Trial 20
PI3K	833.6 ± 10.3^c^	33.69 ± 3.9^b^	6.1 ± 1.03^a^	10.2 ± 1.38^a^	8.05 ± 2.03^a^
P53	0.21 ± 0.003^a^	11.58 ± 1.77^b^	44.16 ± 5.7^c^	15.86 ± 3.88^b^	24.2 ± 7.38^b^
BCl2	165.3 ± 1.59^e^	105.98 ± 14.6^d^	0.89 ± 0.002^a^	54.32 ± 12.3^c^	8.93 ± 0.041^b^
mTOR	850.8 ± 23.19^e^	437.8 ± 16.8^d^	6.35 ± 0.78^a^	157.43 ± 13.78^c^	21.58 ± 1.7^b^
AKT	134.71 ± 8.97^e^	33.45 ± 2.47^c^	0.981 ± 0.02^a^	43.56 ± 1.78^d^	21.3 ± 4.78^b^
PGC1α	209.3 ± 3.12^e^	50.36 ± 12^c^	2.78 ± 0.07^a^	98.3 ± 11.89^d^	9.68 ± 0.1^b^
VEGF	22.3 ± 0.98^d^	8.73 ± 0.08^b^	3.78 ± 0.23^a^	14.65 ± 1.9^c^	7.89 ± 0.08^b^
PTEN	0.125 ± 0.005^a^	22.39 ± 5.71^b^	156.3 ± 3.69^d^	55.69 ± 8.9^c^	43.89 ± 12.3^c^

Data are represented as the mean ± SD. Within the row, means with different letter (a, b, c, etc.) are statistically significant at *p* < 0.05 while means with the same letter are significantly similar at *p* < 0.05. Mean with letter a is the lowest one followed by mean with b, c, etc.

### Western plot

**Figure 4:**
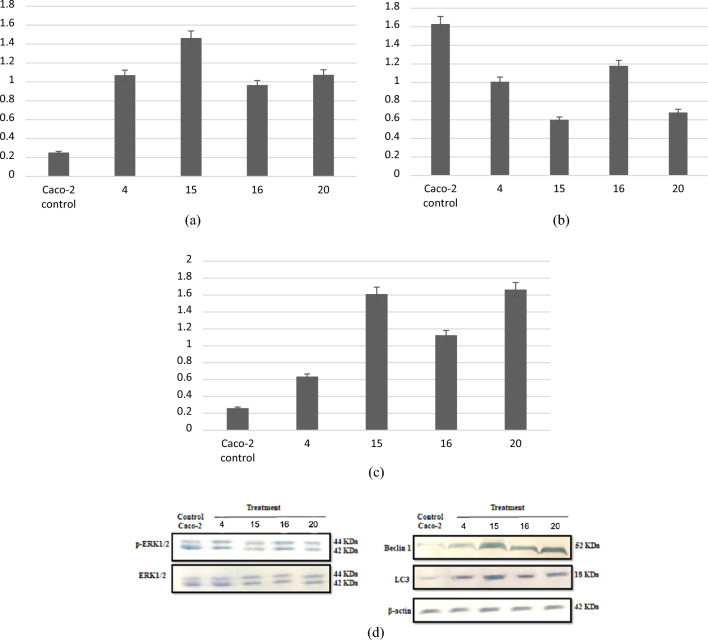
LC3/β-actin (**a**), p-ERK/ERK (**b**), Beclin 1/β-actin (**c**) concentrations and gel electrophoresis of the tested genes (**d**) against the potent trials in Caco-2 cell line.

Erk increased in cancer cells which indicated cell proliferation while autophagy markets LC3 and Beclin decreased in cancer cells. All preparations arrested cellular proliferation by stimulation cell autophagy (Table 4). Results showed that cancer cells had high levels of pErk which indicated tumorigenesis and low level of LC3 and Beclin which indicated autophagy inhibition (Figures S1-S5). The treatment with SER/PRO nanoformula inhibited the PI3K pathway which in turn inhibited mTOR/ERK signaling pathway which consequently activated cell death by autophagy/apoptosis (Fig. 4). The most potent trial 15 (3/2 sericin/propolis ratio for 45 min stirring) was chosen throughout further studies.

**Table 4:** LC3/β-actin, p-ERK/ERK and Beclin 1/β-actin concentrations against the tested trials.

Tested trials	LC3/β-actin	p-ERK/ERK	Beclin 1/β-actin
Caco-2 control	0.2525820636 ± 0.04	1.63024032 ± 0.04	0.2617343287 ± 0.02
4	1.070611277 ± 0.03	1.008833341 ± 0.05	0.6348830018 ± 0.01
15	1.464358362 ± 0.02	0.599475983 ± 0.02	1.612231361 ± 0.03
16	0.965806203 ± 0.02	1.179297501 ± 0.03	1.125361202 ± 0.02
20	1.07405147 ± 0.03	0.678089835 ± 0.02	1.664988302 ± 0.01

Data are represented as the mean ± SD.

### Nanoparticles characterizations

**Figure 5:**
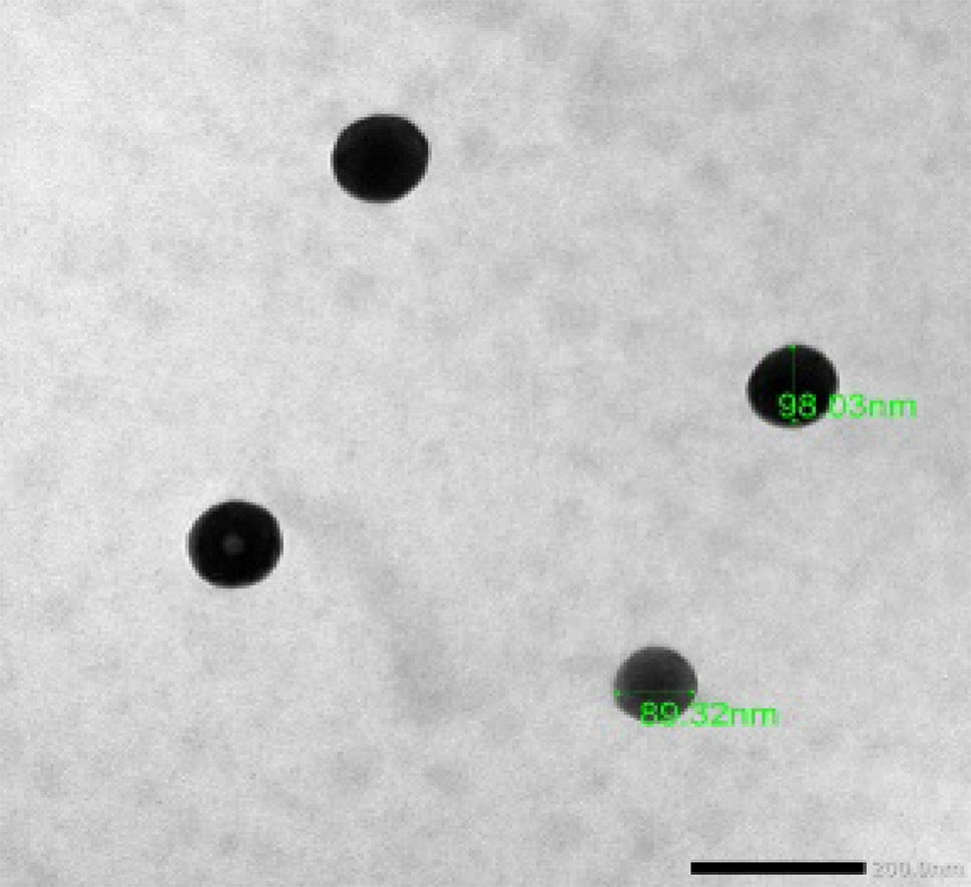
TEM examination of nSE/PRO/FLU.

**Figure 6:**
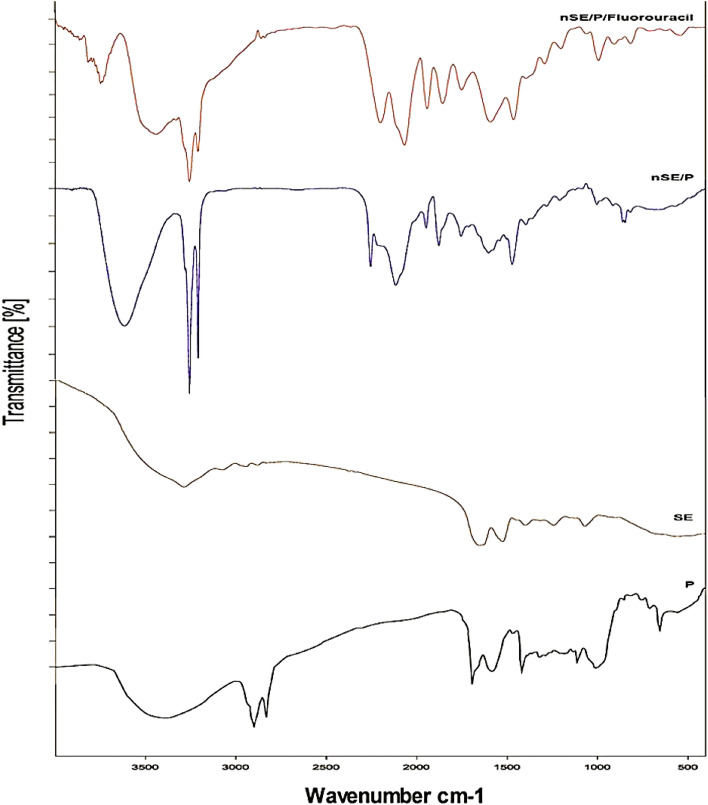
FTIR spectra of propolis (p), sericin (SE), sericin/propolis nanoformula (nSE/P) and sericin/propolis/fluorouracil nanoformula (nSE/P/fluorouracil).

Different fluorouracil concentrations were used to assess the enhanced entrapment efficiency percentage (EE%) (Table 5). It was observed that 10 mg/ml revealed EE% reached 99.2%. While Fig. 5 showed that the formulated nanoparticles were formerly aggregated, well dispersed, and spherical with average size diameter of 95.2 nm. Figure 6 represented the FTIR spectral details of sericin, propolis, and sericin/propolis nanoparticles. Notably, it has been observed that the FTIR spectra of sericin showed peaks in the regions of 3000–3500 cm^-1^ which were associated with N–H stretching vibrations of amide bonds, whereas other distinctive peaks were present at 2924 cm^−1^ indicating C–H stretching. Amide I, II, and III were detected subsequently at 1651, 1522, and 1241 cm^-1^ due to the stretching vibration of the C = O which was significant for determining protein structure. The absorption bands obtained at 1265 cm^-1^ for nSE/PRO was attributed to P = O and P–C stretching. The characteristic peaks of sericin and nSE/PRO that assured the formation of sericin nanoparticles from sericin appeared in the FTIR spectra of nSE/PRO/5-Fluorouracil at 767 cm^−1^ and 1165 cm^−1^ indicating the overlapping peaks of P–O–C and C–O–C. Moreover, other significant characteristic absorption peaks were obtained at 1608, 1431, and 1247 cm^−1^ due to the stretching vibration C = C, C = H, and C = N that indicated the successful incorporation of 5-Fluorouracil and the Sericin/propolis/5-Fluorouracil nanoparticles formation.

**Table 5:** Entrapment efficiency of the formulated nanoparticles with different drug concentrations.

Drug	Drug concentration (mg/ml)	Unentrapped %	EE %
Fluorouracil	10.0	0.800	99.2
	20.0	67.60	32.4
	30.0	85.00	15.0

### Determination of combination index (CI)

Dose Reduction Index (DRI) is defined as the measurement of fold-decrease of an individual drug when used in combinations to achieve a given effect level when compared to the doses of each drug alone. The DRI for Sericin, Propolis, 5-fluorouracil, Sericin/Propolis nanoparticles, Sericin/5-fluorouracil, Propolis/5-fluorouracil and Sericin/Propolis/5-fluorouracil nanoparticles in Caco-2 cell line was calculated using the Calcusyn software (Tables 6, 7). SE and SE/FLU showed higher antitumor activity than propolis alone or the combination between Propolis and 5-fluorouracil. However, nSE/PRO/5-Fluorouracil showed the highest synergistic effect (0.86), with dose reduction index (DRI) of 5-fluorouracil drug reached 1.49 representing the highest antitumor activity. It was then introduced as a promising antitumor nanoformula.

**Table 6:** Determination of combination Index (CI).

Combination trial	CI value	Dose Sericin	Dose Propolis	Dose 5-fluorouracil
Sericin (SE)	ND	1747.95	ND	ND
Propolis (PRO)	ND	ND	2518.12	ND
5-fluorouracil (FLU)	ND	ND	ND	25.8548
Sericin/Propolis (nSE/PRO)	1.37883	1422.62	1422.62	ND
Sericin/5-fluorouracil (SE/FLU)	0.92141	207.532	ND	20.7532
Propolis/5-fluorouracil (PRO/FLU)	0.96371	ND	225.965	22.5965
Sericin/Propolis/5-fluorouracil (nSE/PRO/FLU)	0.86381	178.585	178.585	17.8585

*ND*: not detected.

**Table 7:** Determination of dose reduction Index (DRI) for drug combo.

Combination trial	Dose SE	Dose PRO	Dose FLU	DRI SE	DRI PRO	DRI FLU
SE + PRO	1747.9	2518.12	ND	1.22869	1.7700	ND
SE + FLU	1747.9	ND	25.854	8.4225	ND	1.2458
PRO + FLU	ND	2518.12	25.854	ND	11.1438	1.14419
nSE/PRO/FLU	1747.9	2518.12	25.8548	9.78780	14.1004	1.44776

*ND*: not detected.

### In vivo* investigations*

#### Biochemical, pharmacokinetics and Molecular investigations

**Figure 7:**
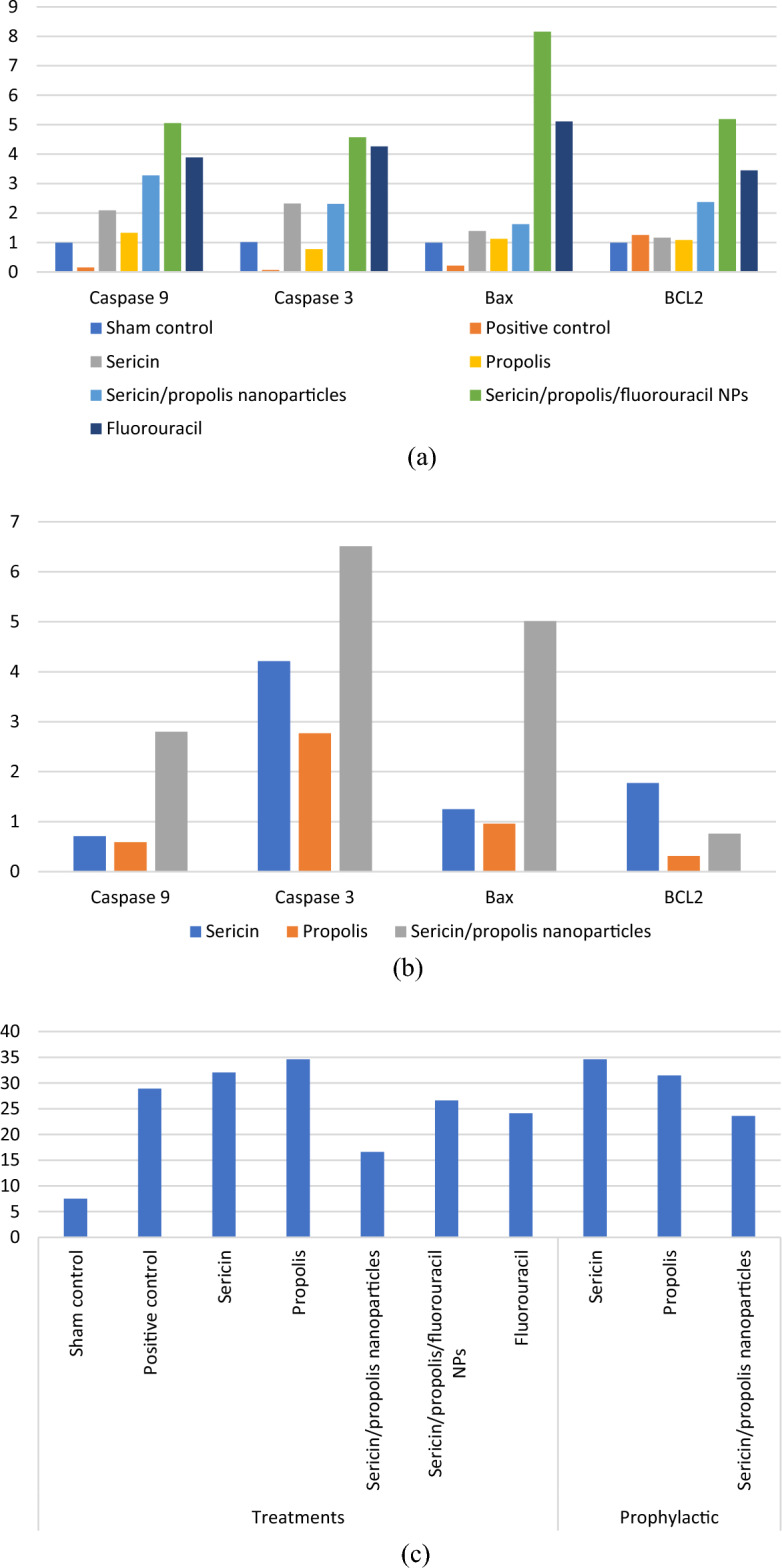
Effectiveness of different treatments (**a**) and prophylactic agents (**b**) on apoptotic markers and TBARS (**c**) in colon tissue.

It is well known that ROS plays a central role during cancer progression where it participates in all cellular events (cell survival, growth, differentiation, protein synthesis and inflammation), therefore Caspase 9, Caspase 3, Bax, BCL2 and TBARS expressions were assessed. It was noticed that sericin/propolis nanoparticles showed potent prophylactic and therapeutic effects against colorectal cancer in vivo (Table 8, Fig. 7).

**Table 8:** Apoptotic markers of colon tissue as affected by different treatments.

Group		Caspase 9	Caspase 3	Bax	BCL2	TBARS
Treatments	Sham control	0.99 ± 0.01^d^	1.01 ± 0.105^c^	0.99 ± 0.02^b^	0.99 ± 0.02^b^	7.52 ± 0.4^a^
	Positive control	0.15 ± 0.02^a^	0.07 ± 0.006^a^	0.21 ± 0.02^a^	1.254 ± 1.6^j^	28.91 ± 2.53^c^
	Sericin	2.09 ± 0.01f.	2.32 ± 0.006^d^	1.39 ± 0.04^e^	1.1611 ± 2.9^i^	32.05 ± 1.93^d^
	Propolis	1.33 ± 0.03^e^	0.77 ± 0.004^b^	1.12 ± 0.02^c^	1.0836 ± 3.4 h	34.6 ± 2.41^d^
	Sericin/propolis nanoparticles	3.28 ± 0.05 h	2.31 ± 0.006^d^	1.62 ± 0.01f.	2.371 ± 1.4f.	16.6 ± 1.65^b^
	Sericin/propolis/fluorouracil NPs	5.05 ± 0.03^j^	4.57 ± 0.003 g	8.16 ± 0.03^i^	5.188 ± 0.05^d^	26.6 ± 1.37^c^
	Fluorouracil	3.89 ± 0.01^i^	4.26 ± 0.001f.	5.11 ± 0.02 h	3.45 ± 0.9^e^	24.1 ± 1.92^c^
Prophylactic	Sericin	0.71 ± 0.03^c^	4.21 ± 0.002f.	1.25 ± 0.05^d^	1.771 ± 0.04^c^	34.61 ± 1.54^d^
	Propolis	0.59 ± 0.02^b^	2.77 ± 0.003^e^	0.96 ± 0.03^b^	0.3135 ± 0.09 g	31.48 ± 1.32^d^
	Sericin/propolis nanoparticles	2.8 ± 0.02 g	6.51 ± 0.005 h	5.01 ± 0.01 g	0.76 ± 0.02^a^	23.58 ± 1.05^c^

Data are represented as the mean ± SD. Within the row, means with different letter (a, b, c, etc.) are statistically significant at *p* < 0.05 while means with the same letter are significantly similar at *p* < 0.05. Mean with letter a is the lowest one followed by mean with b, c, etc.

#### Histopathological findings

**Figure 8:**
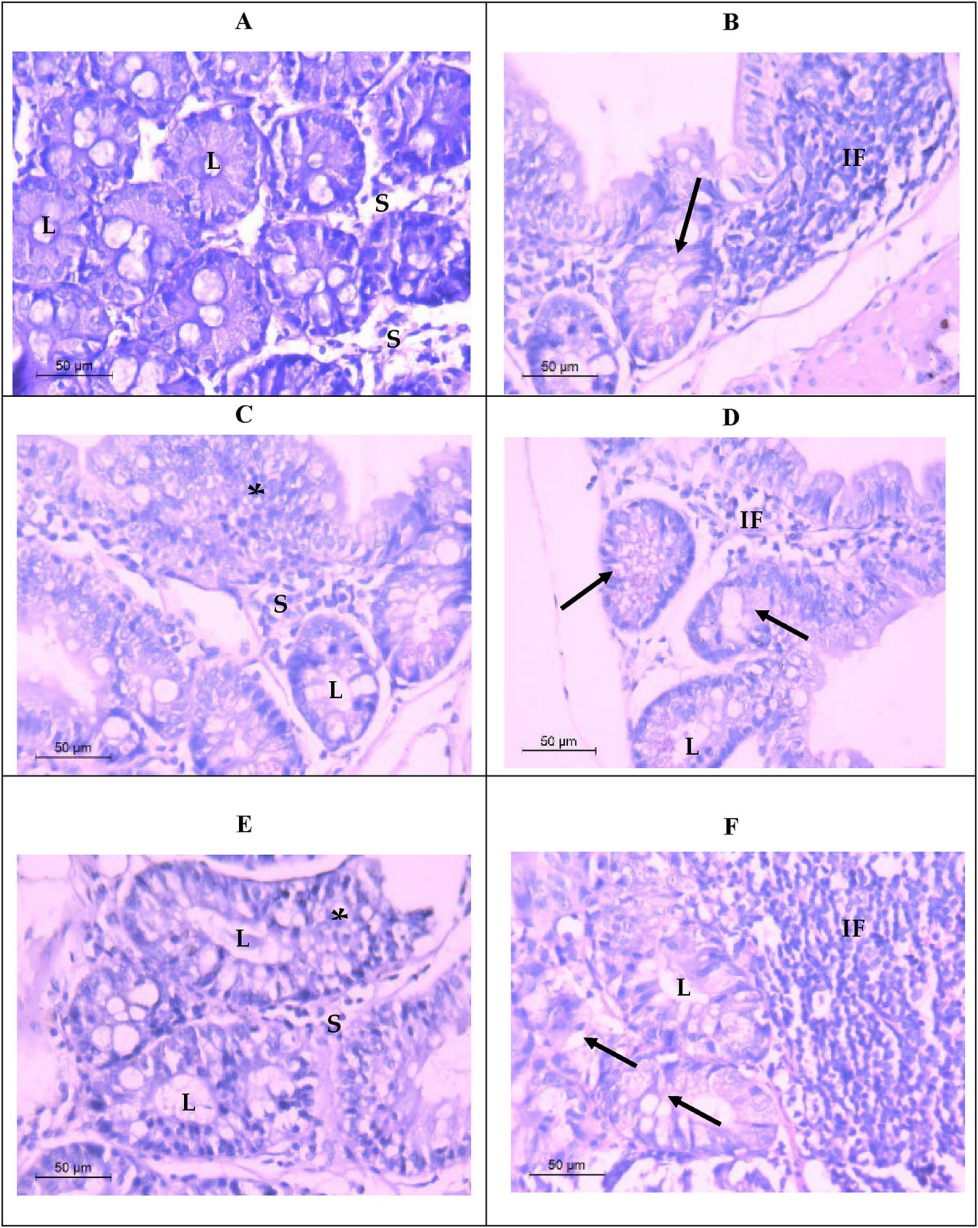

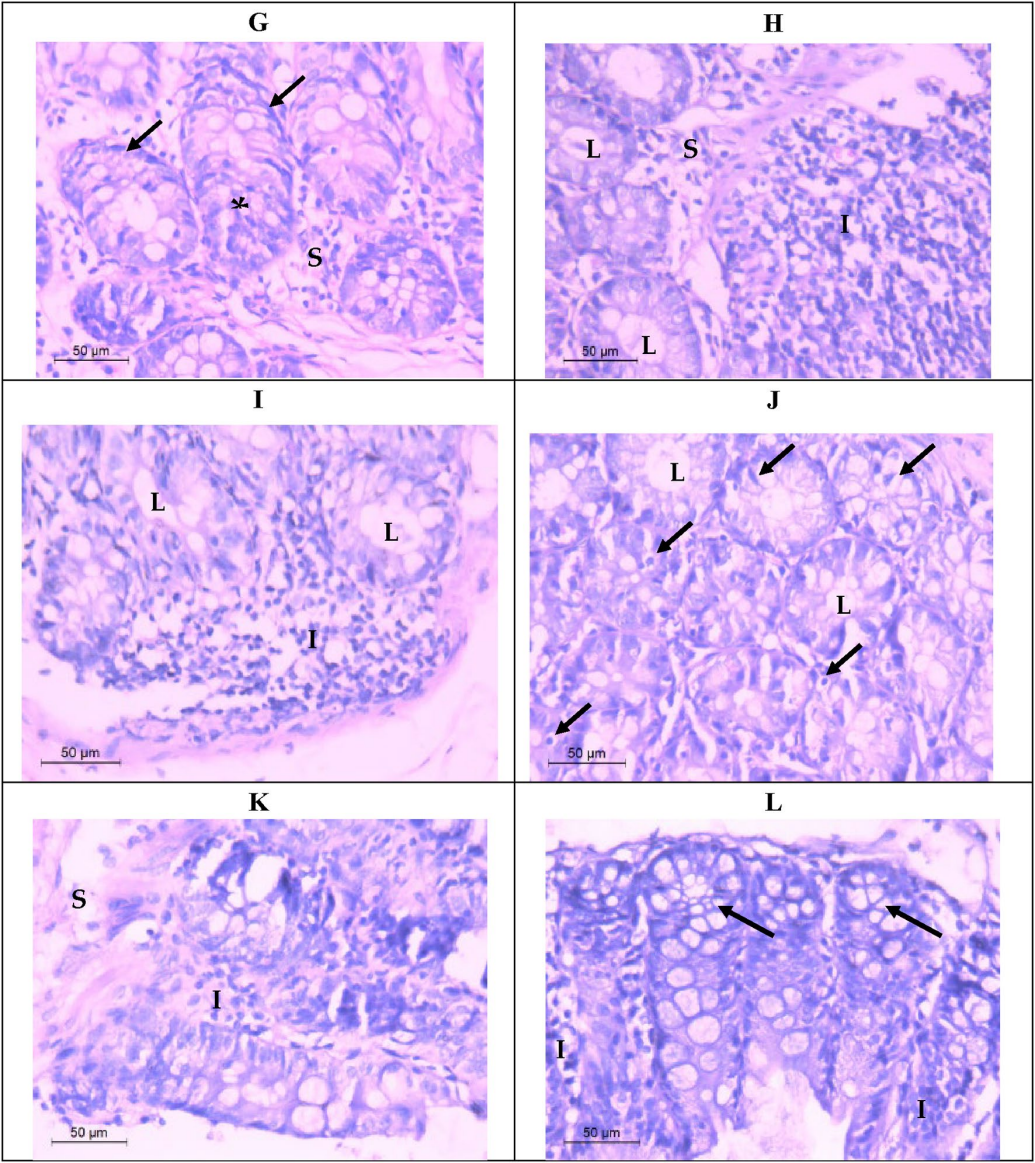
Light micrograph demonstrating H and E stained colorectal sections, (**A**) control group showing normal colorectal glandular tissue with pseudostratified ciliated columnar epithelium enclosing lumen (L), no cell atypia, and stroma (S). (**B**–**D**) positive control group of colorectal tissue with adenocarcinoma showing loss of normal glandular architecture, poor-differentiated adenocarcinoma (arrows), hyperplasia of glandular epithelium (*) with mucinous areas, lumen (L), and abundant tumor-infiltrating lymphocytes (IF). (**E**) illustrating colorectal tissue after applying sericin as a prophylactic showing tubular glandular form with epithelial hyperplasia (*), lumen (L), and stroma (S). (**F**) demonstrating colorectal tissue after applying sericin as a treatment showing poor-differentiated glandular form (arrows), lumen (L), and infiltrating lymphocytes (IF). (**G**) illustrating colorectal tissue after applying propolis as a prophylactic showing epithelial hyperplasia (*) with mucinous areas, dysplasia of the normal glandular form (arrows) with damaged epithelium, and stroma (S). (**H**) demonstrating colorectal tissue after applying propolis as a treatment showing near to normal appearance of glandular epithelium with lymphatic invasion (IF), lumen (L) and stroma (S). (**I**) colorectal tissue after applying nano sericin/propolis as a prophylactic showing numerous tumor-infiltrating lymphocytes (IF) with mucinous areas appearing in the neoplastic epithelium (arrows), damaged epithelium (arrowhead), lumen (L) and stroma. (**J**) colorectal tissue after applying nano sericin/propolis as a treatment illustrating marked nuclear atypia (arrows) and a high nucleus-to-cytoplasm ratio, nuclei were moderately to highly pleomorphic, and lumen (L). (**K**) demonstrating colorectal tissue after applying fluorouracil as a treatment showing necrotic glandular epithelium, lymphocytic infiltration (IF), and stroma (S). (**L**) colorectal tissue after applying nano sericin/propolis/fluorouracil as a treatment illustrating marked multiple lumens (arrows) with mucinous areas, and lymphocytic infiltration (IF).

Normal colorectal tissues have a well-defined organization, with the epithelial cells forming glandular structures and the non-epithelial cells (i.e. stroma) lying in between these glands. Histological sections revealed normal glandular with pseudostratified ciliated columnar epithelium architecture enclosing lumen (Fig. 8A). Histological study results also showed that the administration of the carcinogen (DMH) as a positive control caused loss of the normal acinar architecture and the glandular epithelium differentiation became poor. It must be mentioned that the hyperplasia of the glandular epithelium with mucinous areas was noticed, in addition, there were abundant tumor-infiltrating lymphocytes indicating severe inflammation (Fig. 8B–D). Light microscopy graphs revealed elongated acini because of epithelial cells hyperplasia (Fig. 8E) after applying sericin as a prophylactic agent. The glandular epithelial cells were arranged in a typically stratified configuration. Moreover, colorectal sections after applying sericin as a treatment demonstrated poor-differentiated glandular epithelium and infiltrating lymphocytes (Fig. 8F).

Furthermore, after applying propolis as a prophylactic, colorectal tissue microscopy graphs showed epithelial hyperplasia with mucinous areas, also revealed dysplasia of the normal glandular form. Dysplastic cells showed loss of normal progression from germinal to fully differentiated cells. Marked damage epithelium can be notice in this group indicating apoptosis (Fig. 8G). However, after applying propolis as a treatment, the acinar form of the colorectal tissue was more or less improved. In addition, severe lymphatic invasion appeared indicating inflammation (Fig. 8H).

Although, colorectal tissue after applying nano sericin/propolis as a prophylactic, showed numerous tumor-infiltrating lymphocytes with mucinous areas appeared in the neoplastic epithelium, also, damaged epithelium can be seen (Fig. 8I). While after applying nano sericin/propolis as a treatment, sections demonstrated marked nuclear atypia and a high nucleus-to-cytoplasm ratio. The observed nuclei were moderate to high pleomorphic (Fig. 8J). On the other hand, after applying fluorouracil as a treatment, colorectal sections preformed marked necrosis in the glandular epithelium with lymphocytic infiltration resulting a severe lysis of the acinar form (Fig. 8K). However, after applying nano sericin/propolis/fluorouracil as a treatment the colorectal sections illustrated marked multiple lumens (Fig. 8L). Regardless of the glandular form, the number of lymphocytes infiltrating the stroma was extremely low compared to the other groups (Fig. 8L).

#### Acute toxicity study

**Figure 9:**
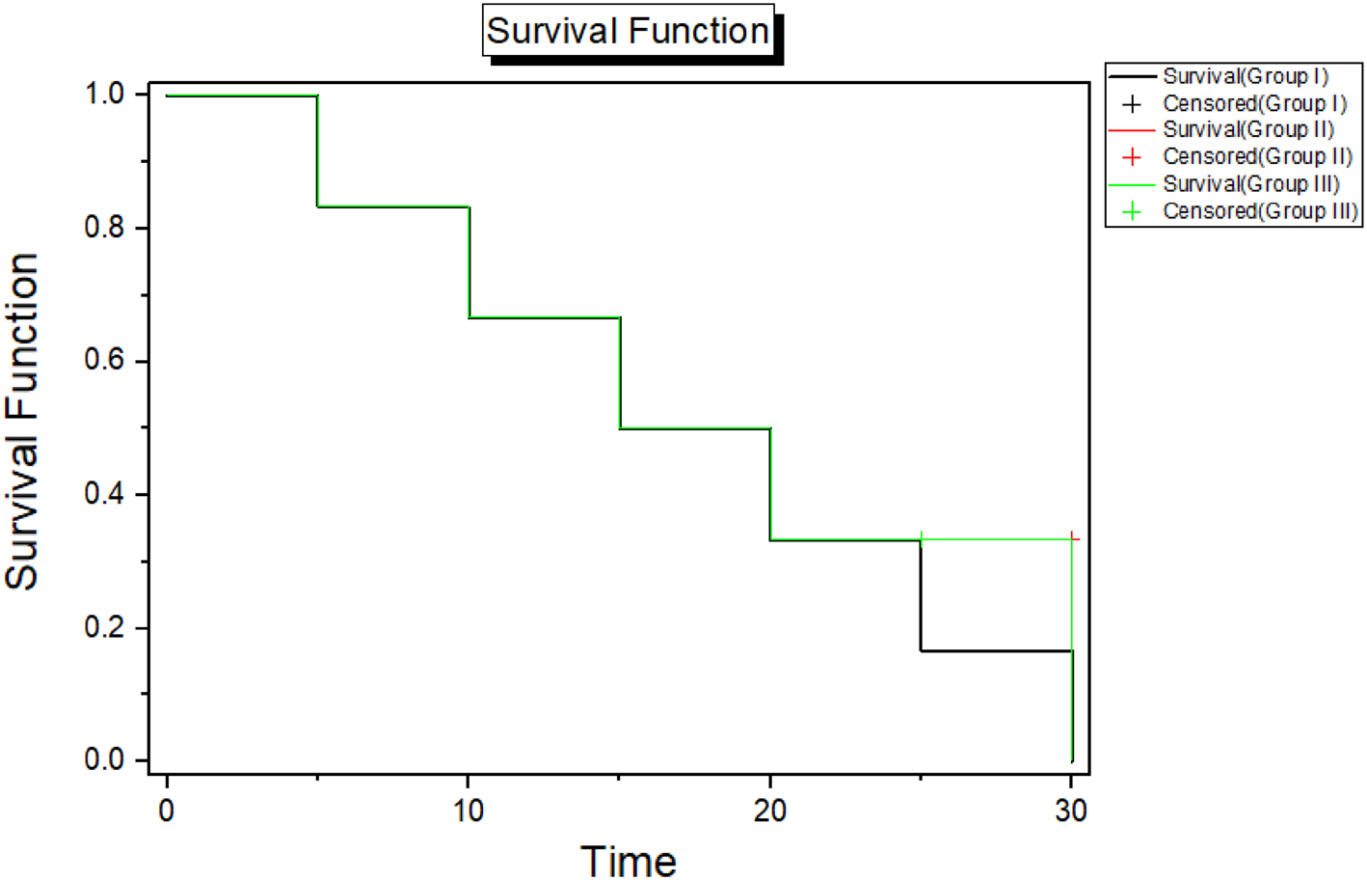
Kaplan–Meier curve assessing the rat’s survival probabilities.

The present investigation aimed to assess the LD50 of sericin/propolis/fluorouracil nanoformula in animal study. Kaplan–Meier method was used to assess the survival rate for 30 days (Fig. 9). It was revealed that LD50 of the prepared nanoformula reached 1 mg/Kg upon oral administration.

#### Pharmacokinetic study

**Figure 10:**
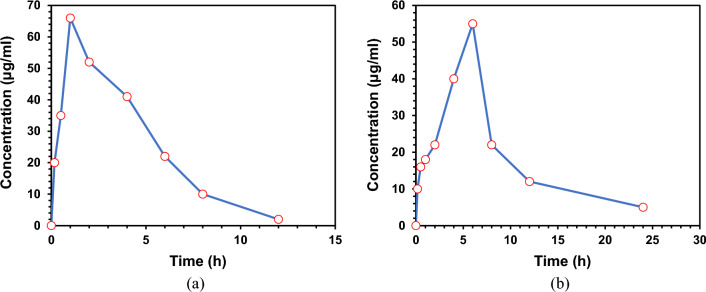
Fluorouracil (**a**) and sericin/propolis/fluorouracil NPs (**b**) concentration Versus time after oral admiration in rats’ plasma.

The present investigation proved the potent effect of the loaded sericin/propolis nanoparticles to maintain a sustainable release of the loaded drug (fluorouracil). Data revealed that the significance difference between the free fluorouracil and loaded sericin/propolis nanoparticles with maximum fluorouracil concentrations in rats’ plasma reached 66 and 55 µg/mL after one hour and 6 h respectively (Table 9 and Fig. 10).

**Table 9:** Fluorouracil (FLU) and sericin/propolis/fluorouracil nanoparticles (NF) concentration in rats’ plasma as affected by time intervals.

Time (h)	FLU (ug/ml)	nSE/PRO/FLU (ug/ml)
0	0.0	0.0
0.2	20.0	10.0
0.5	35.0	16.0
1	66.0	18.0
2	52.0	22.0
4	41.0	40.0
6	22.0	55.0
8.0	10.0	22.0
12.0	2.0	12.0
24.0	0.0	5.0

## Discussion

Our results showed that phosphatidylinositol (PI)-3-kinase/protein kinase/mammalian targeted of rapamycin (PI3K/AKT/mTOR) signaling pathway was active in cancer cells (Table 3). When PI3K was overexpressed it would activate AKT and mTOR in order to stimulate the expression of vascular endothelial growth factor (VEGF) which consequently caused cell angiogenesis and arrested cell autophagy^26,27^. The activation of this signaling pathway is associated with downregulation of its inhibitor Phosphatase and tensin homolog (PTEN). Moreover, cancer cells had upregulated Peroxisome proliferator-activated receptor-gamma coactivator-1 alpha (PGC1α), and B-cell lymphoma 2 (BCL2) that associated with down regulation of P53 that indicated cell proliferation and mitochondrial stress^28,29^. Extracellular signal-regulated kinase (Erk) activates PI3K-mTOR pathway^29^ to promote tumorigenesis, angiogenesis and migration that are associated with inhibition of autophagy and apoptosis^30^. All the prepared treatments in the present study inhibited PI3K/AKT/mTOR signaling pathway and prevented cell proliferation and angiogenesis. It is well known that propolis and sericin inhibit PI3K/AKT and ERK pathway^31,32^.

Moreover, ROS stimulates cell proliferation through the activation of ErK pathway^33^ and stimulates Akt mediated cell survival through inhibition of Bax^34^ and activation of BCL2 which in turn mediated the downregulation of caspases proteins^35^. In agreement with these studies, our results showed that DMH increased ROS production (elevation in TBARS) which induced cell survival (up-regulation of BCL2) and prevented cell apoptosis (downregulation of Bax, Cas9 and Cas 3) through ROS-sensitive mediated pathways which are PI3K/AKT/mTOR and AKT/Erk network (Table 8). According to Suktham et al.^36^, cell viability tests showed that sericin nanoparticles loaded with resveratrol (RSV) significantly slowed the colorectal adenocarcinoma (Caco-2) cells’ growth without affecting the growth of skin fibroblasts. Their in vitro results showed gradually released RSV during 72 h, and drug solubility improvements upon encapsulation which proved their potentiality in pharmaceutical and therapeutic applications.

The treatment and prophylactic effect of both sericin and propolis increased TBARS formation in vivo which activated cell apoptosis through JNK pathways that downregulated BCL2 protein and activated BAX, Cas 9 and Cas 3^35^. While the treatment and prophylactic groups with the potent nanoformula decreased the ROS production which indicated the stimulation of antioxidants system that mainly resulted from PI3K/AKT/mTOR pathway inhibition. This led to FOXO-1 pathway activation that increased the antioxidants production stimulation of autophagy/apoptosis process^35^. Frión-Herrera et al.,^37^ reported that the active component (nemorosone (NEM)) of Cuban propolis arrested the cell cycle in the G0/G1 phase and caused cancerous cell apoptosis, decreasing CRC cell viability and clonogenic capability. Propolis active component (nemorosone) also downregulated BCL2 while elevating TP53 and BAX and activating caspase 3/7. It inhibited cell migration and invasion by boosting E-cadherin, reducing β-catenin and vimentin, and lowering MMP9 activity. It was reported that propolis ethanolic extract inhibited CRC by reducing the aberrant crypt foci (ACFs) in all treatment groups relative to the AOM group^38^. Propolis alone decreased ACFs compared to the control group, suggesting it inhibits CRC initiation or progression, although its therapeutic impact was lower than 5-FLU in that trial. However, different dosages of propolis mixed with 5-FLU substantially decreased ACFs compared to 5-FLU alone, indicating its synergistic impact with 5-FLU. Propolis at 30 and 90 mg/kg reduced ACFs more synergistically than 10 mg/kg, but there was no statistical difference between the two dosages^38^. On the other hand, sericin consumption caused a dose-dependent decrease in the development of colonic aberrant crypt foci and suppressed number of colon tumors^39^. Kaewkorn et al.^40^ reported that the size of the sericin protein may be important for its activity implying that the small sericin had higher anti-proliferative effects than the large sericin (large-size sericin (MW 191,339 kDa) and small-size sericin (MW 61–132 kDa)). Furthermore, they reported that the increased apoptosis of human colorectal cancer SW480 cells was associated with the increased caspase-3 activity and decreased Bcl-2 expression. The anti-proliferative effect of sericin was accompanied by cell cycle arrest at the S phase.

## Conclusions

Sericin/propolis nanoparticles were produced by varying sericin concentrations and stirring durations and the in vitro anticancer (Caco-2 cell line) activity was assessed. Trials 4, 15, 16 and 20 were the most Caco-2-cytotoxic trials. Further investigations were conducted using trial 15 as it was the most potent trial among all (2/3 sericin/propolis ratio and 45 min stirring) with 35.01 IC50, 23.6 zeta potential, 362.9 zeta size and 0.33 PDI. The combination index of the prepared nanoformula revealed that sericin/propolis/fluorouracil nanoparticles showed the maximum synergistic impact (0.86) and chemotherapeutic drug dose reduction index (DRI) reached 1.49. The nanoformula reduced PI3K/AKT/mTOR signaling pathway and stopped cancer cell growth. Sericin/propolis/fluorouracil nanoparticles boosted TBARS production, which stimulated cell apoptosis via JNK pathways that down regulated BCL2 protein and activated BAX, Caspase 9, and Caspase 3. In vivo, the nanoformula inhibited PI3K/AKT/mTOR pathways and activated FOXO-1 pathways to induce autophagy/apoptosis and reducing ROS generation. Histopathological tests confirmed the new nanoformula preventive and therapeutic effects against colorectal cancer.

### Supplementary Information

Supplementary Figures.

## Data Availability

Requested data will be available from the corresponding author upon reasonable request.
