# Supplementary figures and images for: In vitro and in vivo anti-colorectal cancer effect of the newly synthesized sericin/propolis/fluorouracil nanoplatform through modulation of PI3K/AKT/mTOR pathway
Source: Sci Rep. 2024 Jan 29;14:2433. doi: 10.1038/s41598-024-52722-z (PMC10825195; doi:10.1038/s41598-024-52722-z)

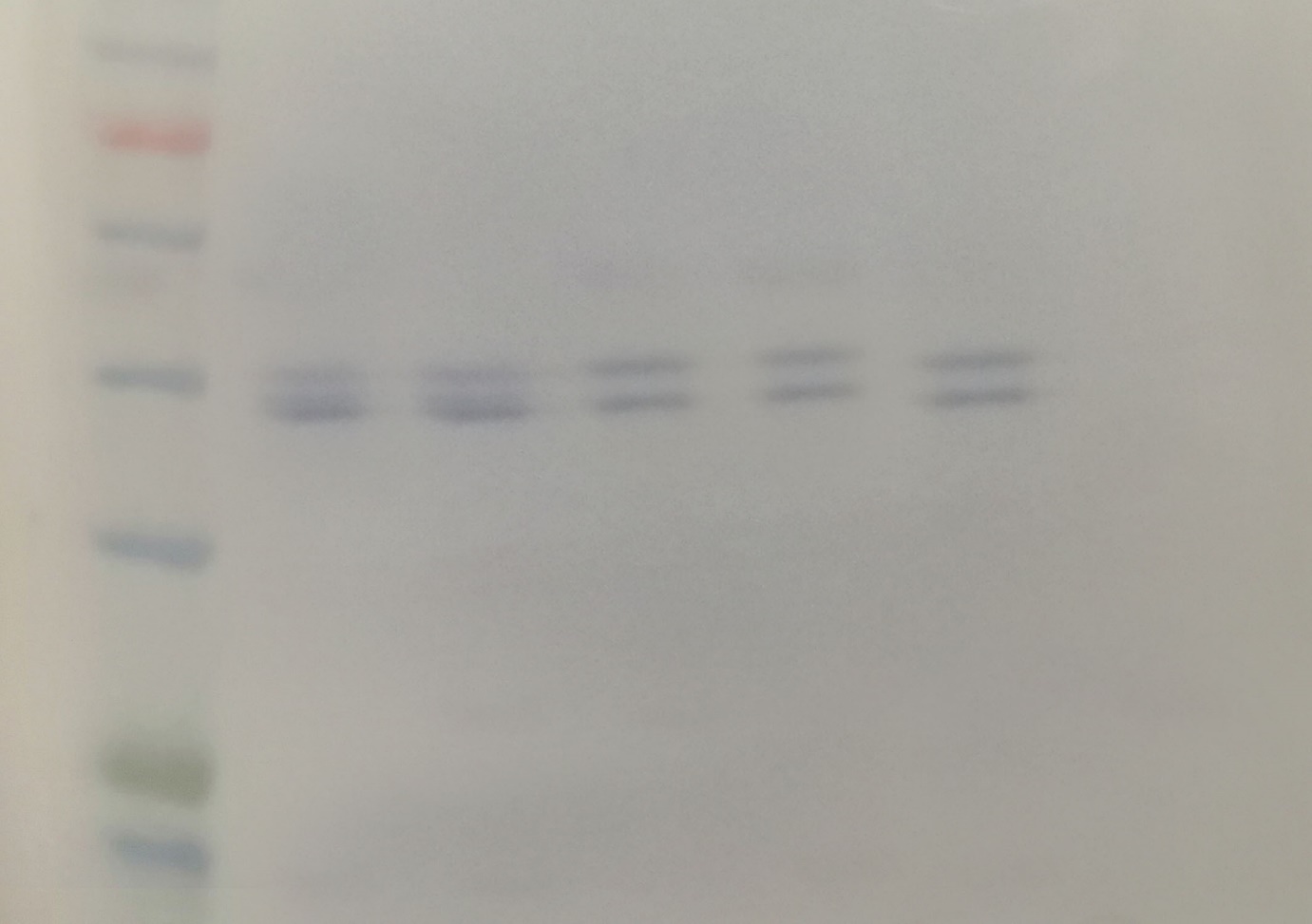


Figure S1. Total ERK.


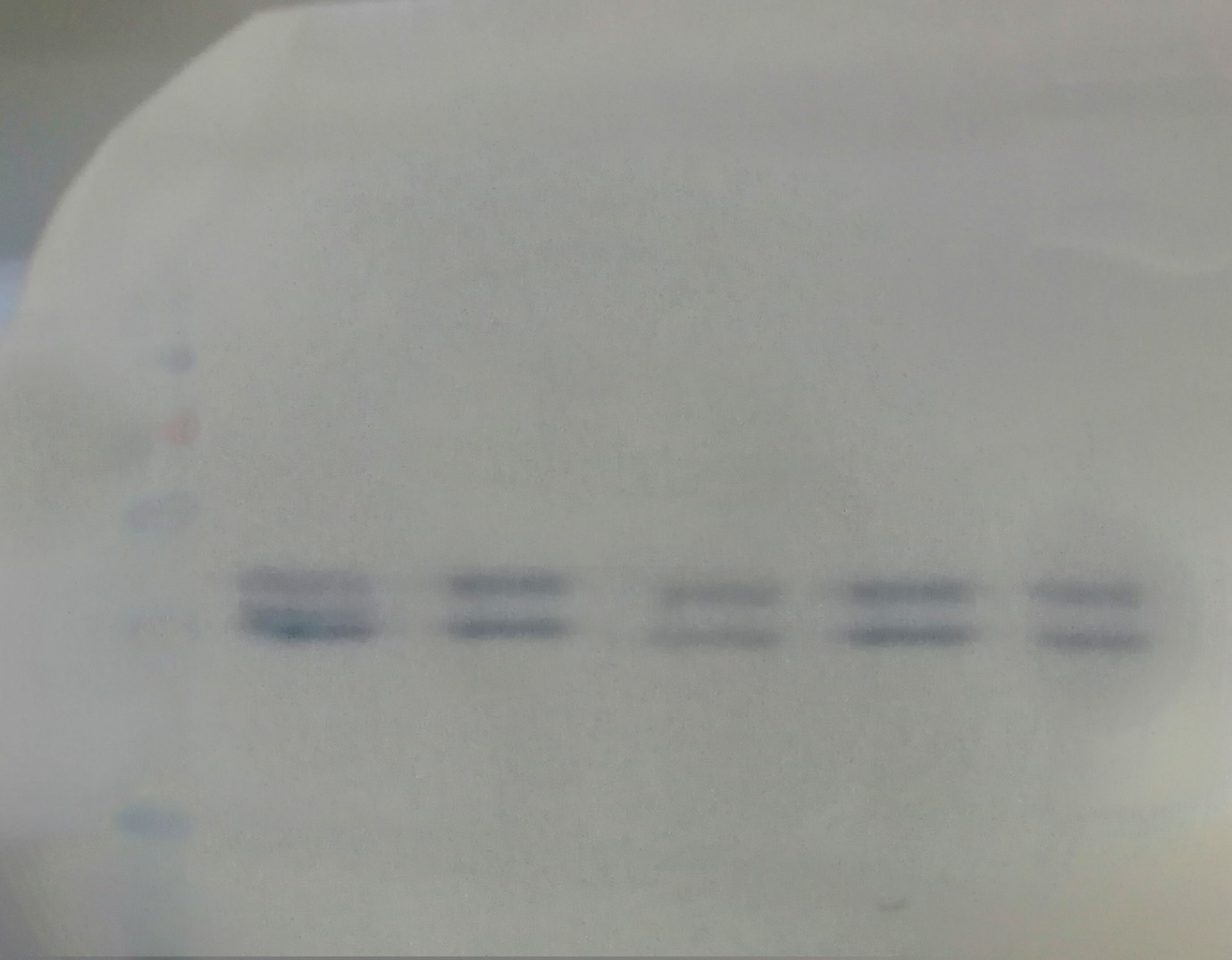


Figure S2. *p*-ERK.


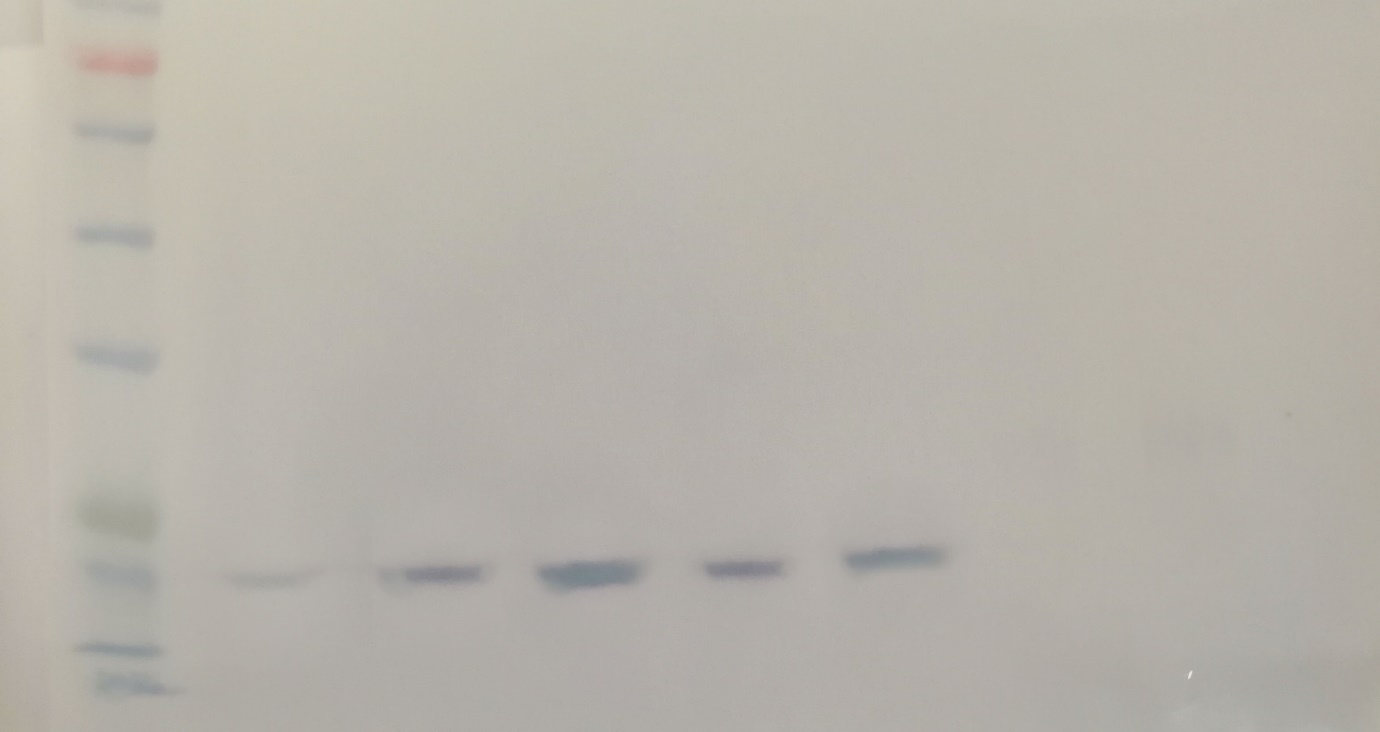


Figure S3. LC3.


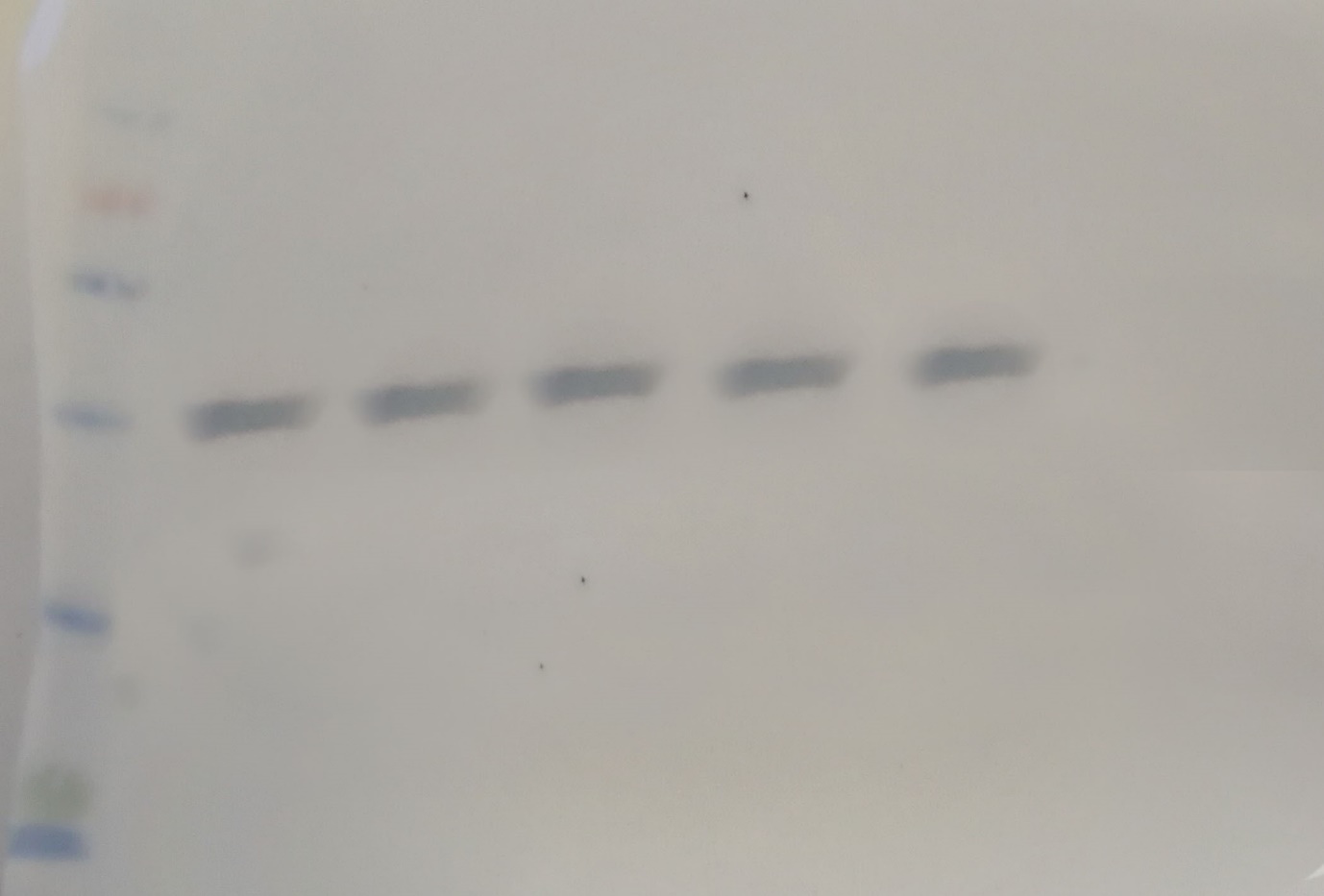


Figure S4. β-actin.


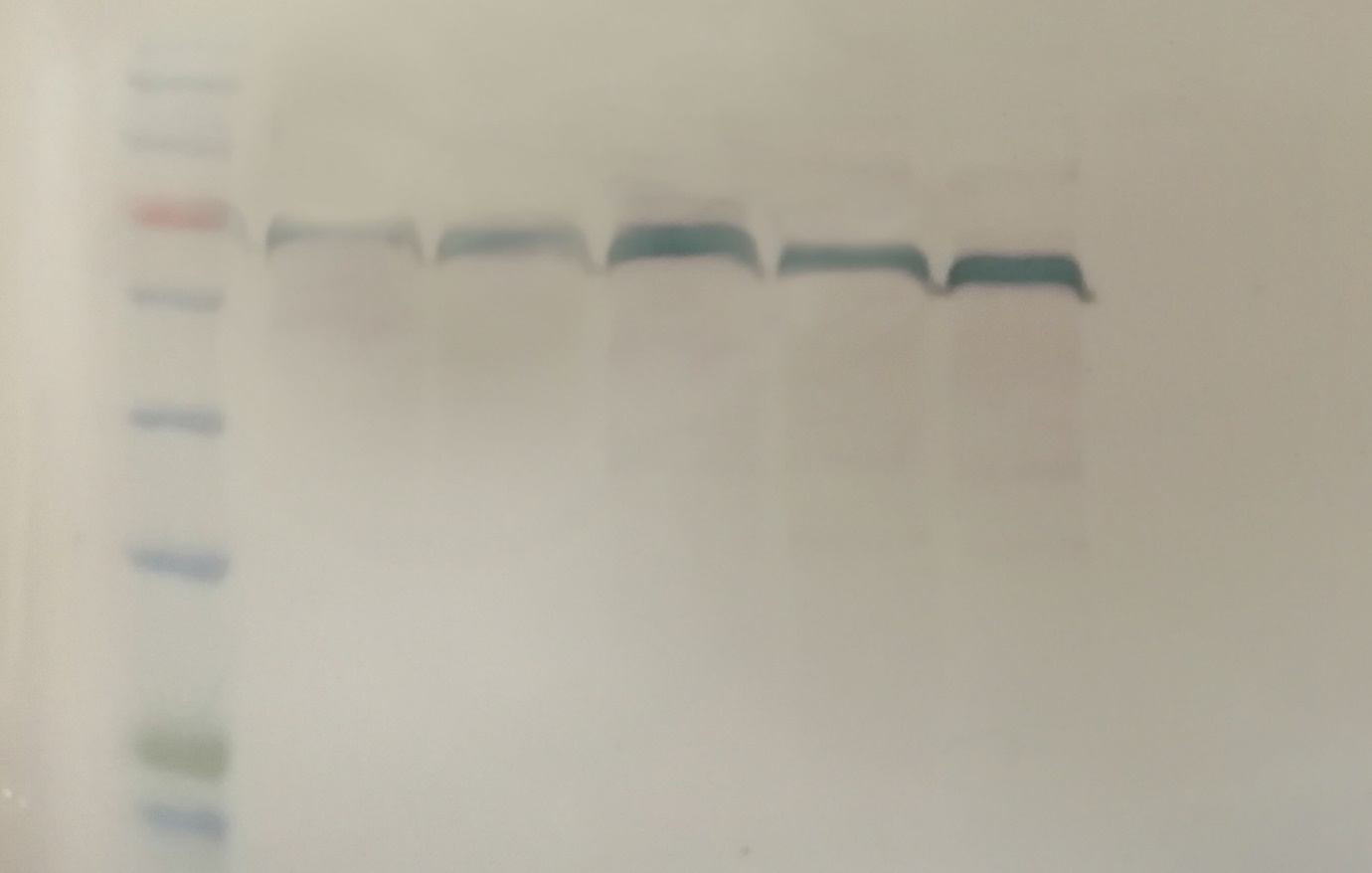


Figure S5. Beclin 1.

Supplement: Supplementary file 1 — Supplementary Figures. [file 41598_2024_52722_MOESM1_ESM.docx]
